# HIV/Tuberculosis Coinfection in Pregnancy and the Postpartum Period

**DOI:** 10.3390/jcm12196302

**Published:** 2023-09-29

**Authors:** Addis Yilma, Hannah Bailey, Petros C. Karakousis, Styliani Karanika

**Affiliations:** 1 Center for Tuberculosis Research, Division of Infectious Diseases, Department of Medicine, The Johns Hopkins University School of Medicine, Baltimore, MD 212875, USA; ayilma2@jhmi.edu (A.Y.); hbaile10@jhmi.edu (H.B.); petros@jhmi.edu (P.C.K.); 2 Department of Molecular Microbiology and Immunology, Johns Hopkins Bloomberg School of Public Health, Baltimore, MD 21205, USA

**Keywords:** TB, HIV, coinfection, pregnancy, postpartum

## Abstract

The convergence of Human Immunodeficiency Virus (HIV) and tuberculosis (TB) represents a considerable global public health challenge. The concurrent infection of HIV and TB in pregnant women not only intensifies the transmission of HIV from mother to fetus but also engenders adverse outcomes for maternal health, pregnancy, and infant well-being, necessitating the implementation of integrated strategies to effectively address and manage both diseases. In this article, we review the pathophysiology, clinical presentation, treatment, and management of HIV/TB coinfection during pregnancy, the postpartum period, and lactation and highlight the differences compared to the general population.

## 1. Introduction

Globally, Human Immunodeficiency Virus (HIV) infection and tuberculosis (TB) pose significant public health challenges [1,2,3]. TB is the primary factor contributing to mortality in people living with HIV/AIDS (PLWHA) [4]. The coexistence of HIV and TB forms a formidable partnership, mutually accelerating the advancement of each other’s detrimental effects [4]. In the absence of appropriate medical intervention, approximately 45% of individuals without HIV who contract TB, and nearly all those who are HIV-positive and afflicted with TB, will succumb to the disease [4]. Disturbingly, 2021 witnessed the loss of approximately 187,000 lives due to HIV-associated TB [4]. In the same year, the global population of pregnant women living with HIV was estimated to be around 1.3 million [5]. A large study including more than 57 million pregnant women indicated that the HIV/TB coinfection cases among pregnant women was 1.9 per one million pregnant women in high and low-burden TB countries. HIV/TB coinfection increases maternal-to-fetus HIV transmission [6] and is associated with adverse maternal and infant outcomes [7]. Interestingly, HIV/TB coinfection in pregnancy is linked to a significantly higher risk of complications compared to those with either HIV or TB infection alone [2,7]. The significant overlap between TB and HIV in pregnancy and the associated poor outcomes underscore the importance of implementing integrated strategies to effectively address and manage both diseases. This review article delves into the pathophysiology, clinical manifestations, outcomes, diagnosis, and treatment pertaining to HIV/TB coinfection in pregnancy, the postpartum period, and lactation.

## 2. Pathophysiology

### 2.1. Immunological Changes during Pregnancy and the Postpartum Period

The immune tolerance of pregnancy plays a critical role in supporting gestation, and it involves substantial alterations in cytokine production and regulation to prevent rejection of the fetus [8,9]. During the different stages of pregnancy, a notable transition occurs from a Th1 pro-inflammatory response to a Th2 anti-inflammatory response, resulting in a shift in cytokine equilibrium [10,11,12], which is instrumental in establishing immune tolerance toward the developing fetus [10].

The suppression of maternal immune responses is believed to be significantly influenced by sex hormones, particularly progesterone and estrogen [8,13,14]. Progesterone plays a crucial role in physiological adaptation during pregnancy by dampening the cytotoxic activity of natural killer (NK) cells and facilitating the proliferation of Th2 cells [8,13,14]. The upregulation of interleukin-10 (IL-10), an anti-inflammatory cytokine, during the second trimester [10,11] suppresses immune reactions directed at fetal antigens and fosters a tolerant environment [11,12]. The third trimester of pregnancy is characterized by elevated levels of pro-inflammatory cytokines, such as IL-6 and IL-1β, which contribute to the development of an inflammatory environment that supports the preparation of the maternal body for labor and delivery [11,12,15]. Additionally, the chemokines CCL2 and CCL3 are required to infiltrate and recruit immune cells into the uterine tissue before labor and delivery [11,12,15]. This inflammatory milieu plays a significant role in ensuring the physiological readiness of the maternal body for the birthing process [12,15]. Estrogens, the other major category of hormones during pregnancy, may regulate the production patterns of Th1/Th2 cytokines [16]. Estradiol (E2) can induce cell-mediated and humoral immune responses [17]. Low E2 concentrations promote Th1 responses and cell-mediated immunity, and high concentrations of E2, as noted in pregnancy, augment Th2 responses and humoral immunity. Estriol (E3) is produced in high concentrations during pregnancy, accounting for almost 90% of all pregnancy-associated estrogens, and it is absent in non-pregnant females [18,19]. The immunological effects of E3 have not been well characterized, although it is likely that its effects are broadly similar to those of E2 since both estrogens signal through the same receptors [20,21].

The postpartum period is marked by a rapid reversal of immunological changes [8,22,23,24]. Following childbirth, the immune system undergoes a process of reconstitution, leading to the restoration of lymphocyte reactivity to its baseline state, from Th2 to Th1, and a resurgence of pro-inflammatory cytokines crucial for robust immune responses [13,16,25,26]. This dynamic shift can potentially serve to reactivate latent infections [27], resulting in exacerbated signs and symptoms, including the reactivation of *Mycobacterium tuberculosis* (*Mtb*) infection [8,28]. In the case of mothers living with HIV, the postpartum modulation of the immune system can also contribute to increased circulating HIV in the bloodstream despite relative stability throughout pregnancy [28,29], potentially exacerbating the clinical manifestations of both diseases in the setting of HIV/TB coinfection.

### 2.2. HIV/TB Coinfection-Related Immune Changes

The HIV-mediated reduction in CD4+ T cells, a primary hallmark of AIDS, increases the risk of primary TB infection, decreases the interval between TB infection and disease, and increases the risk of TB reactivation by as much as 30-fold [30,31]. TB infection in PLWHA can lead to accelerated disease, resulting in heightened symptom severity and adverse outcomes [32]. HIV-1 targets both CD4+ T cells and macrophages, while *Mtb* primarily infects macrophages that rely on the presence of CD4+ T cells for activation and microbicidal activity [8]. It has been shown that there is increased HIV replication at sites of *Mtb* infection in the lungs [33] and within lymphocytes and macrophages in the pleural space [34] of coinfected patients. *Mtb* has been reported to induce HIV-1 replication in chronically or acutely infected T cells or macrophages ex vivo [35,36] as well as in alveolar macrophages and lymphocytes derived from PLWHA [37,38].

## 3. Clinical Presentation

The clinical presentation of HIV/TB coinfection in pregnant and postpartum women varies depending on the HIV stage and TB status (infection vs. disease). Practitioners should be vigilant in recognizing TB-related signs and symptoms to initiate early treatment and improve pregnancy and perinatal outcomes. Although the primary focus of the disease is in the lungs, TB can also involve extrapulmonary sites, including the lymph nodes (e.g., cervical lymphadenitis or scrofula), central nervous system (meningitis or tuberculoma, particularly of the brain stem), bones (mainly the spine), kidneys and multiple organs concurrently (disseminated TB) [39,40,41,42,43,44,45,46,47,48]. Women are less likely to exhibit typical symptoms of pulmonary TB, such as hemoptysis, fever, night sweats, and weight loss [49,50], and these can be further disguised during pregnancy. In addition, pregnant women may experience nonspecific systemic symptoms more likely, including abdominal pain, nausea, vomiting, dysphagia, loss of appetite, diarrhea, chills, and headaches [10]. In a cross-sectional study in South Africa, less than one-third of pregnant women with TB disease manifested fevers or night sweats [51]. The presence of HIV infection further complicates the clinical presentation of TB disease and, depending on the stage of the infection and the level of immunosuppression, may present with isolated lymphadenitis, pharyngitis, myalgias, rash, recurrent infections, night sweats, or persistent fatigue [52] (Table 1).

### Antenatal, Perinatal, and Postpartum Complications

A study analyzing 57 million U.S. inpatient hospital discharges between 2002 and 2014 found that HIV/TB-coinfected pregnant women exhibited significantly higher susceptibility to toxemia of pregnancy, abnormally invasive placenta, and anemia [2]. A retrospective analysis of 7.8 million birth records in U.S. hospitals revealed that pregnant women who were infected with TB had a greater probability of experiencing various complications than those without TB [39]. Notably, a significant proportion of the TB-infected women also had concurrent HIV infection. The observed complications encompassed a range of conditions, such as amnionitis, preterm labor, puerperal anemia, the requirement for blood transfusion, pulmonary infection, acute respiratory distress syndrome, and mechanical ventilation [39]. A case series of 17 pregnant women with HIV/TB coinfection in Karnataka, India, reported that ten had successful term births, four opted for termination of pregnancy, and three experienced fetal demise [53] (Table 1). Another analysis of pregnant women in South Africa, of whom 80 had HIV/TB coinfection and 155 had HIV infection alone, found that the former group had an eight-fold higher incidence of preeclampsia, a significantly greater proportion of coinfected patients required hospitalization during the perinatal period, and the neonates born to coinfected mothers required prolonged hospital stays after delivery and were substantially more likely to be admitted to the neonatal intensive care unit relative to neonates born to mothers with controlled HIV infection alone [54]. These findings highlight the heightened medical challenges pregnant women face with HIV/TB coinfection, emphasizing the need for targeted interventions to address their unique health needs.

## 4. Diagnostics

### 4.1. Screening for TB Infection

The detection of TB infection relies on two types of assays: the tuberculin skin test (TST) and the IFN-γ release assays (IGRAs) [55]. The cutoff for TST differs between individuals without HIV infection (≥10 mm) and PLWHA (≥5 mm), reflecting the increased risk of reactivation in the latter group [56]. For the IGRAs, the cutoff for either QuantiFERON TB Gold In-tube or T-SPOT.TB is independent of HIV status (0.35 IU/mL). The gravid state is not a consideration in determining the cutoff for the TST or IGRAs [13]. Relative to the TST, IGRAs offer higher specificity in the general population and greater sensitivity in pregnant women with HIV/AIDS, although the immunosuppression in the latter group reduces their sensitivity as compared to the general population [55]. In a Kenyan study, 10.3% (8/78) of the pregnant and postpartum women who had consistently positive IGRA results experienced test conversion from TST-negative to TST-positive within six weeks after giving birth [13,57]. HIV diagnosis and older age each negatively affected TST performance compared to IGRAs through pregnancy and the postpartum period, leading to false negative results [13] (Table 2). Similar evidence is provided from two more studies conducted in India, which focused on HIV-infected women during the peripartum period and compared IGRAs with TSTs in a high TB burden setting [58,59]. A higher proportion of women had positive IGRA tests (28%, 71/252) than TSTs (10%, 27/252) during pregnancy and the postpartum period (*p* < 0.005) [59]. Five women developed TB disease during the postpartum period [59]. All five women had a positive IGRA at enrollment, and one had a positive TST [59]. Fifteen pregnant women or women at the postpartum period had a close contact with TB, but the study did not provide the IGRA vs. TST result distribution [59]. The increased specificity of IGRAs relative to TST is attributed to the use of *Mtb*-specific antigens in the former test, which are not expressed by *Mycobacterium bovis* Bacille Calmette–Guérin (BCG) or other environmental mycobacteria. The manufacturing of IGRAs is more standardized, and the test is less technique-dependent than the TST [55]. Unlike the TST, which requires a second visit 48–72 h later to interpret the test result, the IGRAs yield results after a single visit, which is vital for perinatal care. Nonetheless, IGRAs also have certain limitations. These include variability in test results due to specimen handling, higher costs, the need for laboratory infrastructure, and limited availability [55]. Interestingly, the IGRA results have significant within-subject variability, which is essential when interpreting results close to the cutoff points (Table 2) [60].

### 4.2. TB Disease Screening

Significant progress in TB disease diagnosis has been made in the last decade, including the development of highly specific, semi-automated nucleic acid amplification tests known as the Cepheid Xpert^®^ MTB/RIF test and Xpert^®^ MTB/RIF Ultra test (referred to hereafter as Xpert and Xpert Ultra) [61]. These tests detect *Mtb* DNA and provide information on rifampin (RIF) resistance [61]. Notably, Xpert Ultra, with its lower detection limit, offers superior sensitivity compared to Xpert, especially for smear-negative TB cases, which are more common among PLWHA [61]. Given the atypical clinical presentations of TB in pregnancy, WHO recommends a standardized screening tool for pregnant and lactating women consisting of four symptoms: weight loss, fever, ongoing cough, and night sweats [62]. Of note, it is important to consider not only weight loss as a TB screening symptom but also inappropriate weight gain during pregnancy [50]. A study conducted in India found acceptable sensitivity (54.5%) and high negative predictive value (99.3%) of this four-symptom screening tool for diagnosing active TB in HIV-infected pregnant women [63]. However, subsequent studies have shown lower sensitivity (28–50%), underscoring that this screening tool may not capture all cases of TB disease [64,65].

## 5. BCG Vaccine

The BCG vaccine is the only preventative immunization currently in clinical use against TB [48,66]. According to WHO, a single universal dose of BCG vaccine is recommended at birth in settings with a high TB and/or leprosy burden [66], ideally combined with a dose of the hepatitis B vaccine. This recommendation applies to moderate-to-late preterm infants (gestational age > 31 weeks) and low birth weight infants (<2500 g) [66]. According to the Centers for Disease Control and Prevention (CDC), the BCG vaccine in the United States is mainly considered for children with a negative TST who are continually exposed and cannot be separated from adults who are untreated or ineffectively treated for TB disease or have TB caused by strains resistant to isoniazid (INH) and RIF [67]. Because it is a live attenuated vaccine, BCG is contraindicated in pregnancy and PLWHA due to the risk of disseminated BCG disease in the setting of immune compromise [66,67,68,69]. For neonates born to women with an unknown HIV status and neonates with an unknown HIV status born to women living with HIV (regardless of ART status), WHO recommends BCG vaccination if there is no clinical evidence suggestive of HIV infection, as the potential benefits are deemed to outweigh the risks. For neonates confirmed to have HIV infection, it is recommended that BCG vaccination be delayed until ART has been started, and the infant is confirmed to be clinically well and immunologically stable (CD4% > 25%) [66].

## 6. Treatment

### 6.1. ART Considerations

ART plays a crucial role in maternal health and the prevention of perinatal HIV transmission [70,71]. In 2018, WHO updated its guidelines for the first-line treatment of pregnant adults, replacing efavirenz (EFV) with dolutegravir (DTG) [72]. A randomized clinical trial conducted subsequently at 22 different sites to compare the safety and efficacy of DTG-based vs. EFV-based ART regimens in pregnant women living with HIV confirmed superior viral suppression at the time of delivery with DTG-based regimens, further supporting the WHO recommendation to use DTG in all populations [73] (Table 3). The same study highlighted that the composite adverse pregnancy outcome (occurrence of preterm delivery, small for gestational age, stillbirth, or spontaneous abortion) was observed in significantly fewer women assigned to DTG/emtricitabine (FTC)/tenofovir alafenamide (TAF) compared with women assigned to either DTG/FTC/tenofovir disoproxil fumarate (TDF) or EFV/FTC/TDF, suggesting that it may be preferable to initiate TAF, which has lower renal and bone toxicity [74,75,76,77], rather than TDF during pregnancy (in combination with DTG and FTC) (Table 3). Of note, this was the first clinical study showing the safety and efficacy data of TAF during pregnancy [73]. The updated US guidelines published in 2023 for pregnant women living with HIV have embraced this evidence, listing TAF as a first-line drug in pregnancy and TDF as an alternative drug, which is associated with more adverse pregnancy outcomes [78] (Table 3). Additionally, the US guidelines have incorporated the WHO recommendation regarding DTG as a first-line drug in pregnancy with the caveat that this may not be an option in women previously taking carbotegravir as HIV pre-exposure prophylaxis [78]. When combined with DTG, TAF/FTC has been reported to cause more treatment-emergent obesity in non-pregnant adult women than TDF/FTC. However, the weight gain associated with the former regimen may be beneficial in pregnancy [73,79] (Table 3).

### 6.2. TB Infection: Treatment Considerations

For pregnant women with well-controlled HIV on ART, treatment for TB infection can be delayed until 2–3 months postpartum to minimize possible adverse drug reactions. For women at high risk for progression from TB infection to TB disease, especially those who have had recent contact with someone with infectious TB, preventive treatment should not be delayed based on pregnancy alone, even during the first trimester [80,81]. The treatment options for TB infection in pregnant women living with HIV are generally the same as those for pregnant women who do not have HIV infection, considering potential drug–drug interactions and overlapping medication toxicities. The available regimens include a 4-month daily regimen of RIF, a 3-month daily regimen of INH and RIF, or a 6- or 9-month daily regimen of INH, with the addition of pyridoxine (vitamin B6) supplementation to avoid peripheral neuropathy [82]. Due to lack of safety data during pregnancy, the 3-month weekly INH and rifapentine regimen is not recommended for pregnant women [81]. Although INH is not teratogenic in animals or humans and can be co-administered with any ART regimen, INH-induced hepatotoxicity might occur more frequently in pregnancy and postpartum [83]. For this reason, monthly monitoring of liver transaminases during pregnancy and the postpartum period is recommended [80]. Although RIF is not teratogenic in humans, because of a potentially increased risk for RIF-related hemorrhagic disease among neonates born to women receiving anti-TB therapy during pregnancy, prophylactic vitamin K, in a single 10-mg dose should be administered to the neonate following delivery [65,68]. Due to its induction of the cytochrome P450 system, RIF interacts with many drugs, including some first-line ART agents. When co-administered with DTG, the DTG dose should be doubled to 50 mg twice daily [84,85]. Raltegravir (RAL) may also require dose adjustment [86]. The combination of TAF and RIF has not been studied in human studies to confirm pharmacokinetics and efficacy. TAF, unlike TDF, relies on drug transporters that RIF induces, and there is a concern that TAF levels may be decreased, affecting antiviral efficacy [87]. Based on adversely affecting ART pharmacokinetics, RIF is contraindicated with protease inhibitors [88,89], etravirine [90,91,92], doravirine [93], rilpivirine [94], bictegravir [95], cabotegravir [94,96] and elvitegravir/cobicistat [97].

### 6.3. Isoniazid Preventive Therapy

A clinical trial of INH preventive therapy (IPT) among HIV-infected women in high TB prevalence settings was recently conducted, randomly assigning women to receive IPT for 28 weeks, either initiated during pregnancy or at week 12 after delivery, and mothers and infants were followed through week 48 after delivery [98]. The combination of IPT and ART proved effective in reducing the risk of HIV-associated TB. However, the early IPT initiation group showed a higher incidence of adverse pregnancy outcomes compared to the deferred treatment group without additional benefits related to TB risk or maternal/infant death. Importantly, however, none of the women were household contacts of TB cases; most were IGRA-negative and receiving EFV-based ART [98]. In the United States, IPT is recommended for pregnant women with HIV and those who are household contacts of a TB case [99]. However, for pregnant women receiving effective ART and those who are not household contacts of TB cases, initiation of IPT may be deferred until after delivery.

### 6.4. Drug-Sensitive and Drug-Resistant TB Disease: Treatment Considerations

In contrast to treatment for TB infection, treatment for active TB should not be postponed in pregnant women living with HIV, regardless of ART use and immune status. If drug-sensitive TB is suspected, the preferred initial treatment regimen is INH, RIF, and ethambutol (EMB) daily for two months, followed by INH and RIF daily or twice weekly for seven months (for a total of nine months of treatment), as per CDC guidelines [81]. Although EMB is teratogenic in animal models, the doses at which this toxicity is observed are much higher than those used in the clinical setting, and EMB-related teratogenicity has not been reported in humans [100,101]. EMB-related optic neuropathy has been reported in adults [101,102], but changes in visual acuity have not been detected in infants born after exposure in utero. The CDC does not recommend using pyrazinamide (PZA) in pregnancy because its effect on the fetus is unknown [81]. In contrast, because PZA is not teratogenic in animals [103], WHO includes this drug in its recommendations for routine use in pregnant women [104]. If PZA is not included in the initial treatment regimen, the minimum duration of TB therapy is 9 months [78,80,99]. The decision regarding whether to include PZA for TB treatment should be made after consultation among obstetricians, TB specialists, and patients and after consideration of gestational age and PZA susceptibility testing of the infecting strain [81,99].

Evidence for using the majority of the second-line drugs for TB during pregnancy is limited, and a TB expert should be consulted if multi-drug-resistant TB in pregnancy is suspected [78,80,99]. In a recent South African study, the use of bedaquiline in pregnancy was linked to an increased incidence of low infant birth weight [105]. Ethionamide has been associated with teratogenicity in some animal models and central nervous system defects in humans [106]. However, there have been case reports where ethionamide use did not lead to similar concerns, and the consensus is that this drug may be used if necessary [107]. Aminoglycosides/polypeptides carry a risk for ototoxicity [108,109,110] if administered during pregnancy. No safety data exist for cycloserine. Although fluoroquinolones have not been associated with an increased risk of congenital disabilities or musculoskeletal abnormalities [111], their use should be avoided in pregnancy and children unless other safe options are unavailable [99].

### 6.5. Breastfeeding

Effective ART during pregnancy and the postpartum period decreases the risk of HIV transmission through breast milk to less than 1% [78,112,113,114]. US-based HIV practice guidelines advise mothers who are not virologically suppressed to use formula or pasteurized donor human milk [78,112,113,114]. Since 2010, WHO guidelines recommend breastfeeding plus ART given to mothers and infants, which is supported by evidence from many randomized controlled trials [115,116,117,118,119]. WHO guidelines that prioritize breastfeeding over formula feeding are grounded in the elevated risk of gastrointestinal infections associated with the use of contaminated water in low- and middle-income countries [120,121]. The current recommendation from WHO states that mothers living with HIV should breastfeed for at least 12 months and may continue breastfeeding for up to 24 months or longer while being fully supported for ART adherence [120]. Regarding TB treatment, breastfeeding should not be discouraged for women being treated with first-line TB drugs (INH, RIF, EMB) because the concentrations of these drugs in breast milk are too low to cause adverse reactions to the nursing newborn [122,123]. Importantly, for the same reason, drugs in breast milk are not an effective treatment for TB disease or TB infection in infants [81,122,123].

### 6.6. Newborn HIV and TB Management Considerations

All newborns with perinatal exposure to HIV should receive ART during the neonatal period to reduce the risk of perinatal HIV transmission with selection of the appropriate regimen guided by the level of transmission risk. The level of risk is defined based on the mother’s ART receipt, duration and adherence during pregnancy, mother’s HIV viral load at certain time points, the diagnosis of acute HIV infection during pregnancy and the presence of a positive newborn HIV virologic test [124,125,126]. In the low-transmission risk setting, ART prophylaxis with zidovudine (ZDV) is recommended [127,128] while on the high-transmission setting, presumptive HIV therapy using either ZDV, 3TC, and NVP (treatment dose) or ZDV, 3TC, and RAL is recommended [129,130,131]. Infants born to a parent with TB should undergo medical evaluation, TST, chest X-ray, three gastric aspirates on three consecutive days and examination of the placenta for pathology and AFB smear and culture. In infants suspected of having congenital TB disease, treatment is considered with INH, RIF, PZA, and an injectable agent if hospitalized, independently of the test results [132,133].

## 7. Conclusions and Future Directions

Given the unique pathophysiological changes during pregnancy and the postpartum period, there are special considerations for managing HIV/TB coinfection in this population. This review offers crucial clinical insights into the formidable public health challenges presented by HIV/TB coinfection. It underscores the grave implications of this dual infection, particularly for pregnant women, where HIV/TB coinfection not only heightens the risk of transmitting HIV from the mother to the fetus but also leads to detrimental adverse outcomes for both. The increased risks and complications linked to HIV/TB coinfection during pregnancy, postpartum, and lactation necessitate further research on identifying universally applicable, cost-effective, diagnostic and treatment strategies and proactive interventions to provide optimal care. Further research is also needed to identify the immunological responses during HIV/TB coinfection in the context of pregnancy and the postpartum period. These collaborative endeavors will enhance outcomes and the overall welfare of pregnant women and infants facing HIV/TB coinfection.

## Figures and Tables

**Table 1:** Manifestations and complications during pregnancy and HIV/TB coinfection.

Disease	Clinical Manifestations/Complications
Pregnant women with TB disease	chest discomfort, hemoptysis, fever, night sweats, weight loss (or not appropriate weight gain), and fatigue
Pregnant women with TB/HIV coinfection	hemoptysis, fever, night sweats, and weight loss (not appropriate weight gain), isolated lymphadenitis, pharyngitis, myalgias, rash, recurrent infections, persistent fatigue
Pregnant women with HIV/TB infection complications	anemia, abnormally invasive placenta, amnionitis, preterm labor, pulmonary infection, sepsis, acute respiratory distress syndrome, and mechanical ventilation, preeclampsia, fetal demise

**Table 2:** Differences between Tuberculin Skin Test and IFN-γ release assays.

Tuberculin Skin Test	IFN-γ Release Assays
Cutoff without HIV infection is ≥10 mm and with HIV infection is ≥5 mm	No cutoff for HIV infection
Affected negatively by HIV/AIDS and age	Higher sensitivity and specificity in pregnancy with HIV/AIDS
Technique dependent	Not technique dependent
Two visits to report results	Single visit to report results
Trained personnel for interpretation	Test variability
Low cost	Higher cost
No need for laboratory infrastructure	Need for laboratory infrastructure
Widely available	Not readily available

**Table 3:** Treatment considerations per ART drug in pregnancy.

ART Drug	Treatment Considerations
Dolutegravir (DTG)	First-line treatment in pregnancy Superior viral suppression at the time of delivery compared to EFV-based regimens When RIF is co-administered with DTG, the DTG dose should be doubled Not an option for women taking cabotegravir as HIV pre-exposure prophylaxis
Efavirenz (EFV)	Not part of preferred regimens during pregnancy
Tenofovir alafenamide (TAF)	First-line drug in pregnancy, proven safe and efficacious Associated with fewer adverse pregnancy outcomes compared to TDF-based regimens Lower renal and bone toxicity than TDF in conjunction with DTG or EFV When combined with DTG, TAF/FTC cause more treatment-emergent obesity in non-pregnant adult women than TDF/FTC. However, the weight gain may be beneficial in pregnancy Relies on drug transporters that RIF induces; TAF levels may be decreased, affecting antiviral efficacy
Tenofovir disoproxil fumarate (TDF)	TDF is an alternative drug to TAF in pregnancy Associated with more adverse pregnancy outcomes than TAF-based outcomes Does not rely on drug transporters that RIF induces
